# Supplementary material for: Effects of Glucose and Corn Syrup on the Physical Characteristics and Whipping Properties of Vegetable-Fat Based Whipped Creams
Source: Foods. 2022 Apr 20;11(9):1195. doi: 10.3390/foods11091195 (PMC9102422; doi:10.3390/foods11091195)
Supplement: Supplementary file 1 [file foods-11-01195-s001.zip › foods-1659777-supplementary.pdf]

Supplementary Materials

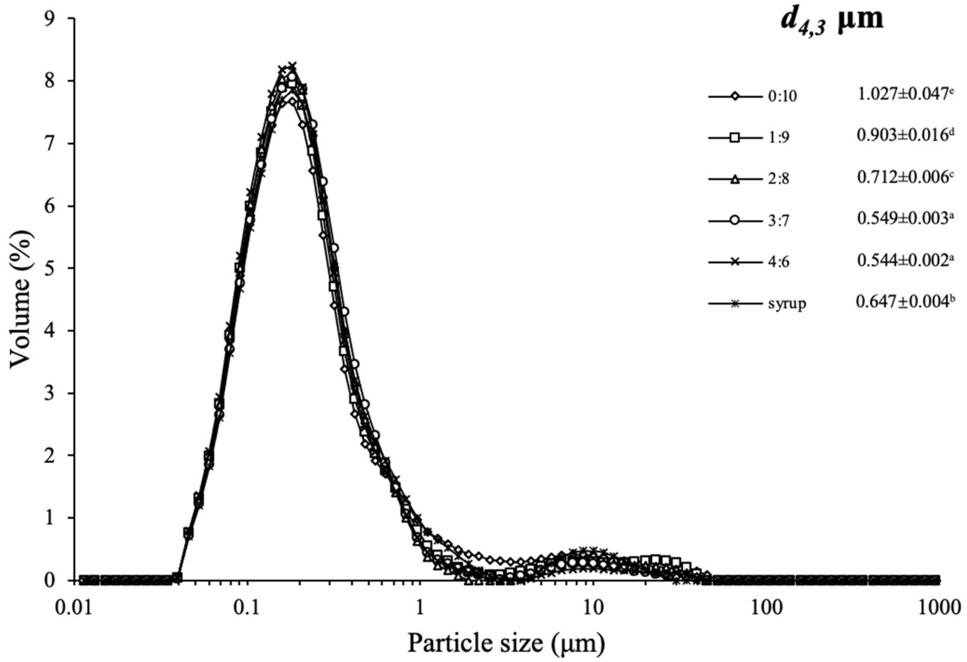

Figure S1. Particle size distribution of the emulsions with 30 wt% sugar concentrations at different ratio between maltodextrin and glucose (0:10, 1:19, 2:8, 3:7, 4:6). (Syrup represents the emulsion with 30 wt% corn syrup. Different letters of a-e indicate significant differences ($p < 0.05$) among different ratios of MDX and glucose.

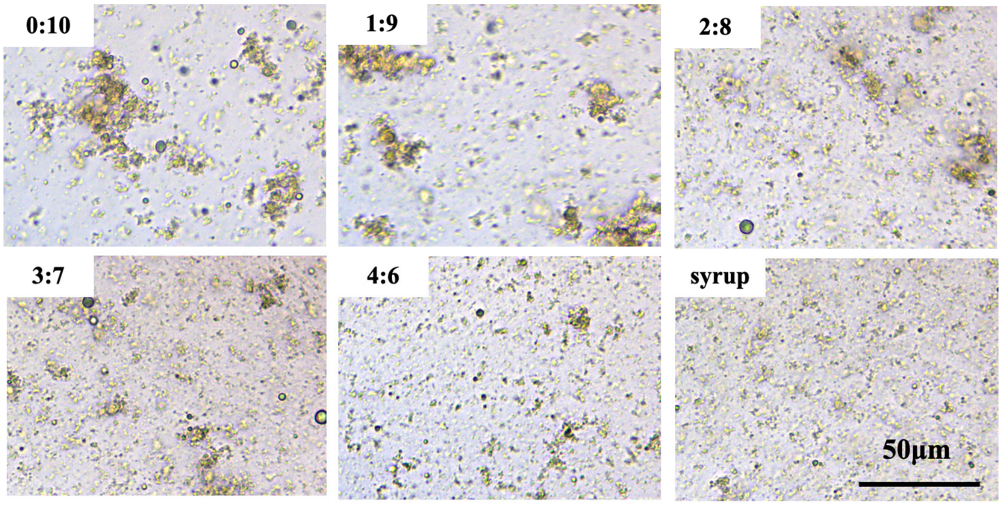

Figure S2. Microstructure of the emulsions with 30 wt% sugar concentrations at different ratio between maltodextrin and glucose (0:10, 1:19, 2:8, 3:7, 4:6). (Syrup represents the emulsion with 30 wt% corn syrup).

Table S1. Interfacial protein content and SSA of whipped creams with addition of different sugars at different concentrations

sample	Interfacial protein content (mg/g)	SSA (m^2/g)
Creams-glucose	10.38±0.25[a, x]	3.82±0.03[e, x]
	15.54±0.71[b, x]	3.68±0.06[d, x]
	18.72±0.65[c, x]	3.54±0.01[c, x]
	21.25±0.37[d, x]	3.21±0.06[b, x]
	21.39±0.04[d, x]	2.96±0.04[a, x]
Creams-corn syrup	15.88±1.05[a, y]	18.56±0.06[e, y]
	22.26±0.19[b, y]	15.50±0.07[d, y]
	22.59±0.80[b, y]	13.66±0.08[c, y]
	23.77±0.41[b, y]	10.69±0.06[b, y]
	26.02±0.21[c, y]	8.19±0.06[a, y]

[1] Different letters of a-e indicate significant differences ($p < 0.05$) among different sugar concentrations. Different letters of x, y indicate significant difference ($p < 0.05$) between glucose and corn syrup in the same proportion.
